# Strong coupling between quasi-bound states in the continuum and molecular vibrations in the mid-infrared

**DOI:** 10.1515/nanoph-2022-0311

**Published:** 2022-08-09

**Authors:** Kaili Sun, Min Sun, Yangjian Cai, Uriel Levy, Zhanghua Han

**Affiliations:** Shandong Provincial Key Laboratory of Optics and Photonic Devices, Center of Light Manipulation and Applications, School of Physics and Electronics, Shandong Normal University, Jinan 250358, China; Department of Applied Physics, and the Center for Nanoscience and Nanotechnology, The Hebrew University of Jerusalem, Jerusalem, Israel

**Keywords:** bound state in the continuum, strong coupling, vibrational resonance

## Abstract

Strong light–matter coupling is of much interest for both fundamental research and technological applications. The recently studied bound state in the continuum (BIC) phenomenon in photonics with controlled radiation loss rate significantly facilitates the realization of the strong coupling effect. In this work, we report the experimental observation of room temperature strong coupling between quasi-BIC resonances supported by a zigzag metasurface array of germanium elliptical disks and the vibrational resonance of polymethyl methacrylate (PMMA) molecules in the mid-infrared. Based on the approach of tuning the quasi-BIC resonance by changing the thickness of the coated PMMA layer, we can easily observe the strong coupling phenomenon, manifested by significant spectral splitting and typical anti-crossing behaviors in the transmission spectrum, with the spectral distance between the two hybrid photon-vibration resonances significantly larger than the bandwidth of both the quasi-BIC resonance and the PMMA absorption line. Our results demonstrate that the use of quasi-BIC resonance in all-dielectric nanostructures provides an effective and convenient approach for the realization of strong coupling effect.

## 1 Introduction

The strong coupling between optical resonances and matter excitations provides an effective path of controlling light–matter interactions, profoundly changing various behaviors of light and matters. In the strong coupling regime, the matter excitation and the optical resonance interact through the near field with the coherent energy exchange at a rate higher than the original decay rates of both, resulting in a new pair of polaritonic states with anti-crossing behavior and energy separation by Rabi splitting [1, 2]. These new hybrid states inherit the properties of both light and matters, suggesting potential applications like room temperature Bose–Einstein condensation [3], threshold-less lasing [4] and even single-photon switches [5], among others. So far, this fascinating phenomenon has been observed in many different types of excitations, such as excitons in two-dimensional materials [6] and semiconductors [7], electronic spins in nitrogen-vacancy centers [8], or even cyclotron transitions in two-dimensional electron gas [9], etc. Strong coupling between the vibrations of molecules and various micro-nano structures including plasmonic resonators [10] and optical microcavities [11, 12] has become a focus of broad attention thanks to the so-called collective coupling where a large number of material oscillators can couple to one single optical mode, enabling an effectively higher coupling efficiency. Strong or even ultra-strong coupling between phonons and photon/plasmon excitation can be easily achieved in these systems [13, 14].

A key parameter characterizing the coupling effect is the energy exchange rate *g* between light and matter [15]. In order to observe strong coupling, *g* should be larger than both the photon leakage rate of the optical resonator, *κ*, and the nonradiative loss rate for the excitation transition, *γ*. For passive optical resonators, *g* is a product of both the transition dipole momentum of the matter and the vacuum field amplitude of the resonator [16], while the latter is reversely proportional to the mode volume of the resonator. So in order to realize the strong coupling effect, one should either reduce *κ*, or increase *g*, or achieve both. To this end, one can either reduce the mode volume of the resonator, or employ an optical system with high quality (Q) factors. One typical example of achieving the strong coupling is to make use of plasmonic resonators, which have the advantages of ultra-small mode volumes and large local field enhancement. Although successful demonstrations have been widely reported on the strong coupling between plasmonic nanostructures and molecular J-aggregates [1], the large dissipation losses associated with metallic structures lead to the drawbacks of low resonance Q factors (large *κ*), which challenge the observation of the strong coupling effect. Alternatively, one can use all-dielectric resonating structures exhibiting ultra-high Q factors, including photonic crystal cavities [17], whispering gallery [18] or Fabry–Perot resonators [19]. Unfortunately, those devices are relatively bulky with a fairly large mode volume (typically from a few to a few tens of wavelength scale), leading to a relatively low coupling strength and preventing the occurrence of the strong coupling effect at the nanoscale, which is more attractive and interesting. An ideal optical system to realize the strong coupling effect should possess both large Q factors and ultra-small mode volumes simultaneously. For periodic structures, a discussion of the mode volume can be found in [20].

In recent years, the concept of bound states in the continuum (BIC) and quasi-bound states in the continuum (QBIC) have attracted significant attention due to their ultra-high Q factors with huge electric field enhancement [21, 22]. Their applications have surged into many fields, including molecular fingerprint retrieval [23, 24], biosensing [25, 26], thermal emitters [27, 28] and enhanced nonlinear harmonic generations [29–31]. The BIC phenomenon, initially proposed in quantum mechanics by von Neumann and Wigner in 1929 [32], was extended to optical systems in 2008 [33]. Since then, scientists have explored various photonic systems to investigate the realization of QBIC resonances with ultra-narrow bandwidth [34]. Such QBICs are usually supported by all-dielectric nanostructures with in principal no dissipation loss while the radiation loss is suppressed either due to symmetry incompatibility or by a destructive interference between different radiation channels [35]. As a result, QBIC resonances in dielectric or semiconductor nanostructures exhibit ultra-high Q factors while maintaining moderate mode volumes and therefore are extremely favorable for enhancing light–matter interactions, especially for facilitating the realization of strong coupling at the nanoscale. Although the strong coupling between QBIC modes and excitations have been reported either numerically [36, 37] or experimentally [38] in the optical band, there are few reports on the strong coupling phenomena based on the QBIC resonance in the mid-infrared (MIR), where molecules exhibit many remarkable excitation behaviors like the rotational and vibrational resonances. The easy processing of these molecules at the large scale and the possibility of changing many chemical or material properties of various organic materials makes the strong coupling between optical resonance and vibrations in the MIR even more interesting [39].

In this paper, we report and experimentally demonstrate to the best of our knowledge for the first time room temperature strong coupling in the MIR between a QBIC resonance and a vibrational resonance of molecules. The QBIC resonance is supported by a zigzag array of germanium disks on a calcium fluoride (CaF_2_) substrate. After the fabrication of the Ge disk array on the CaF_2_ substrate, a thin layer of polymethyl methacrylate (PMMA) molecules was subsequently spin-coated on top of the array to provide the vibration resonance. Our experimental measurements of the transmission spectrum demonstrate a well-pronounced Rabi splitting phenomenon, which agrees quite well with the numerical results revealed by the finite element method (FEM) technique.

## 2 Structure and results

**Figure 1:**
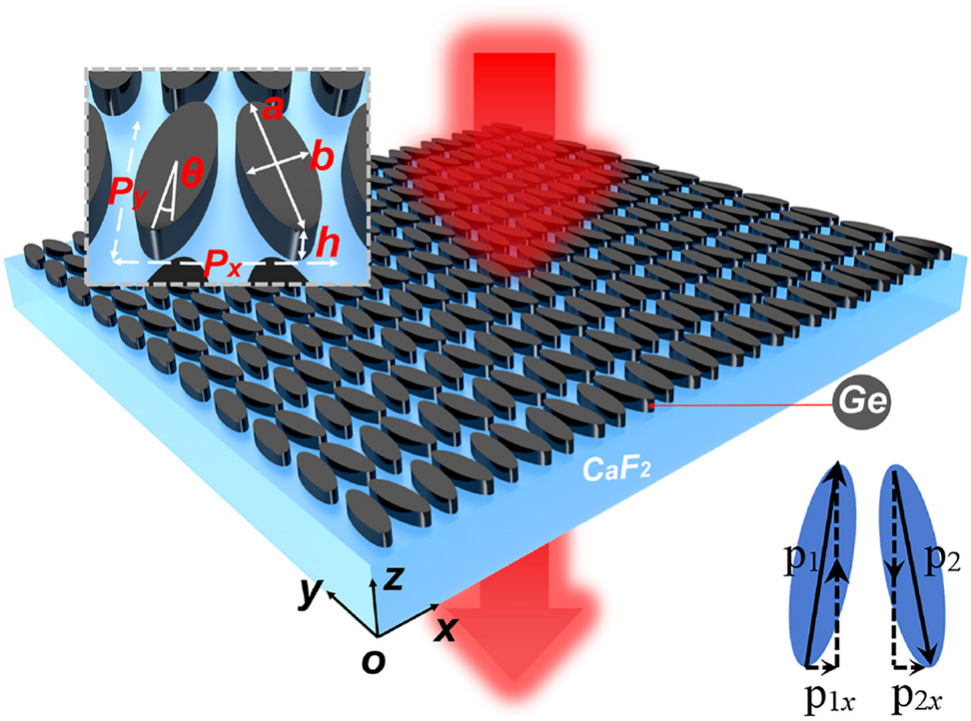
Schematic diagram of the structure supporting the QBIC resonance, with the inset presenting an enlargement of a single periodic cell. The inset in the lower right corner denotes the formation of the QBIC mode due to two counter-directional electric dipoles.

Figure 1 shows a schematic diagram of the investigated metasurface structure supporting the QBIC mode in this paper. The metasurface is composed of a zigzag array of Ge elliptical disks on the CaF_2_ substrate. Both Ge elliptical disks within one unit cell have a relative tilting angle *θ* from their major axis (i.e. the angle between the two disks is 2*θ*). The QBIC mode is attributed to the excitation of two electric dipoles supported in individual disk with counter-directional dipole momentums (see the inset of Figure 1). When the long axis of the elliptical disks is in parallel, the structure is in a perfectly symmetric state exhibiting an ideal BIC mode with no coupling to the external environment and therefore cannot be excited by a plane wave. Such BIC originates from the spatial symmetry incompatibility between the mode distributions of the structure’s eigen resonance and the incident plane wave. However, when a certain rotation is introduced into the disk pair, the symmetry is broken enabling a resonance transition from the ideal BIC to a QBIC mode, which can be easily excited by a simple linearly polarized plane wave. The *x* component of the electric field in the incident plane wave is critical to excite the QBIC mode, when the two electric dipoles have a non-zero net component of the dipole momentum. In our FEM calculations, the material parameters of CaF_2_ are fitted by using the tabulated experimental data [40], and its dielectric constant is described by the formula:

(1) $$\begin{array}{ll} \varepsilon_{CaF_{2}} & =1.33973+\frac{0.69913\lambda^{2}}{\lambda^{2}-0.09374^{2}}+\frac{0.11994\lambda^{2}}{\lambda^{2}-21.18^{2}}\\ & \;+\frac{4.35181\lambda^{2}}{\lambda^{2}-38.46^{2}} \end{array}$$

where *λ* is the wavelength. The inset in Figure 1 shows a local magnification of the metasurface, where *h* = 300 nm is the height of the Ge disks, *P*_*x*_ = 3 μm and *P*_*y*_ = 3.5 μm are the array period, and *a* = 3.35 μm and *b* = 1.03 μm represent the long and short axis of the elliptical disks, respectively. The two Ge disks within one unit cell have a center-to-center distance of 1.4 μm. All the geometrical parameters are carefully designed, giving rise to the QBIC resonance close to the vibrational resonance of PMMA around 5.78 μm. The red arrow in Figure 1 indicates the power flow of mid-infrared (MIR) light whose transmission spectrum is used for optical characterization. All simulations and experimental results of this study are obtained for the *x*-polarized excitation under normal incidence. Due to the introduction of *θ*, the structure can be excited by a simple linearly polarized plane wave to produce the symmetry-protected QBIC mode. Importantly, the bandwidth of this QBIC highly depends on the asymmetry of the structure, thus providing an effective way of controlling the Q factor and resonance wavelength using geometrical parameters.

**Figure 2:**
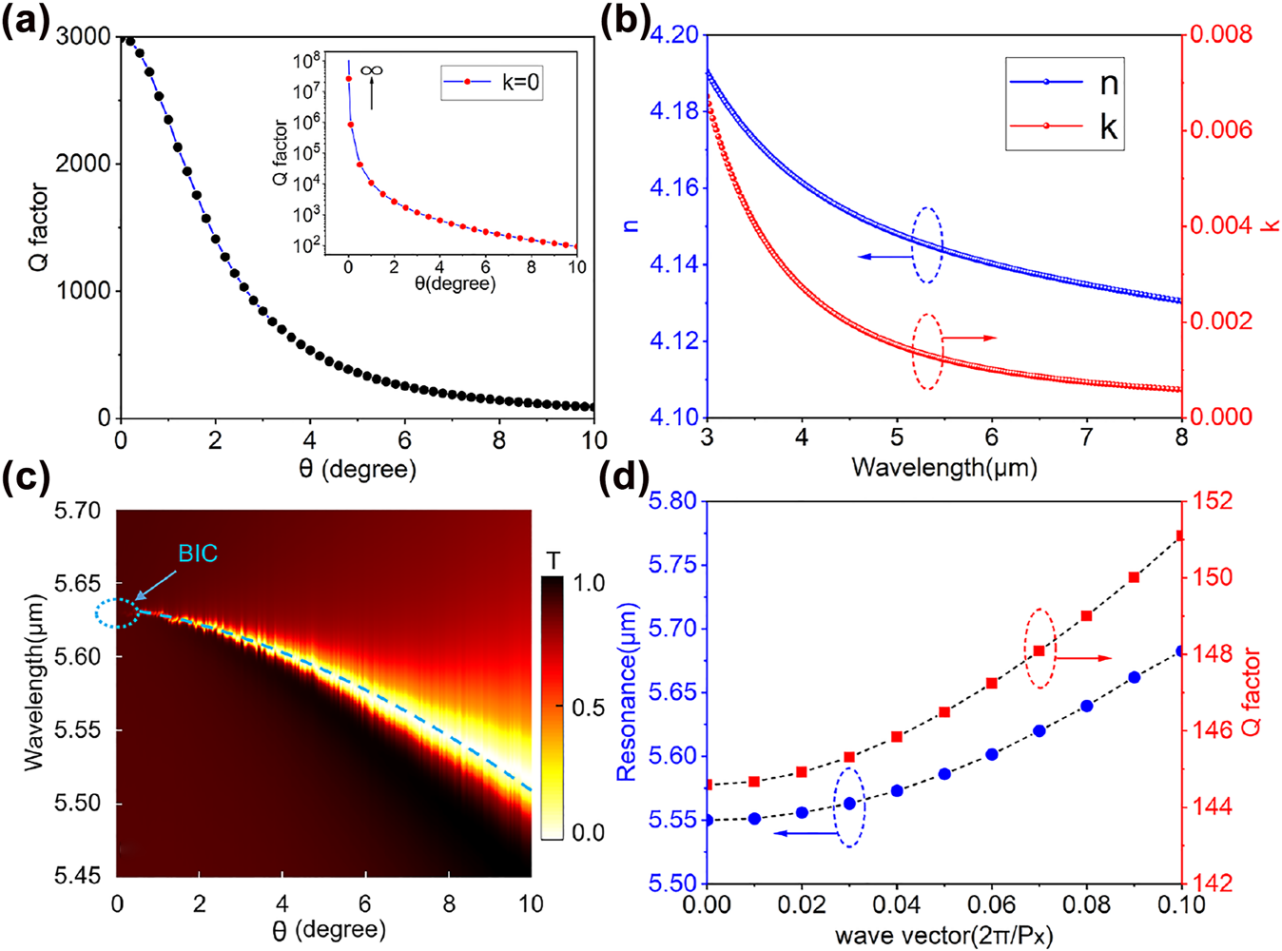
Numerical results of QBIC resonance properties. (a) Dependence of the Q factor of the QBIC resonance on the tilting angle between the elliptical disks. The black solid dots present the results when the material absorption of amorphous Ge (using the *k* value in Figure 2(b)) is considered, while the inset is for the case when Ge is assumed to be lossless. (b) The refractive index of the 300 nm Ge thin film measured by a MIR ellipsometer. (c) Simulated transmission spectrum as a function of *θ*, the blue dashed line representing QBIC dispersion, and the blue dashed circle marking the BIC mode at *θ* = 0. (d) The resonance frequency of the structure and the associated Q factor as a function of the transverse wave vector when *θ* = 8°.

The properties of the QBIC modes supported by the metasurface structure, including both the resonance frequencies and the corresponding Q factors, are evaluated using the eigen-frequency analysis (implemented in a commercial software of Comsol Multiphysics), combining the Floquet period boundary conditions in the lateral directions with perfectly matched layers (PML) in the *z*-direction. The transmission spectra are calculated by using S parameters with the port boundary conditions. We first assume the Ge to be crystalline and lossless [41]. In this case, the dependence of resonance Q factor on the asymmetric parameter *θ* is presented in the inset of Figure 2(a). It is well-known that when the structure is strictly symmetric (*θ* = 0) and no dissipation losses are considered, the structure works in an ideal BIC mode and cannot be excited by plane waves, exhibiting a zero bandwidth [23]. The dependence of the Q factor on the asymmetric parameter *θ* is consistent with the typical inversely quadratic behavior of the symmetry-breaking designs [22]. A lossless Ge layer on CaF_2_ can be achieved by first growing a crystalline layer of Ge on a silicon substrate using e.g. molecular beam epitaxy (MBE) technique, followed by a bonding to the CaF_2_ substrate and a subsequent removal of Si. In this work, in order to simplify the experiment, a 300 nm-thick of amorphous Ge is deposited directly onto the CaF_2_ substrate by electron beam evaporation (EBV), which results in a certain level of dissipation loss compared with single-crystal Ge. The complex refractive index of the deposited Ge material is measured by an ellipsometer working in the MIR (see Methods) and the results are presented in Figure 2(b). When the real material parameters are taken into account in the numerical simulations, the dependence of the QBIC Q factor on *θ* is shown by the black solid points in Figure 2(a). Clearly, the absorption loss of the amorphous Ge from EBV has a significant influence on the resonant bandwidth, and when *θ* = 0, the Q factor is found to be 3000 instead of infinity. This is also the result of some roughness in the fabricated structure. Here, although the radiation loss is eliminated, the overall Q factor is determined by the contributions from the material absorption and roughness. However, we will demonstrate in the following part that even when the material absorption is considered, the strong coupling between the QBIC modes and the vibrational modes can still be steadily observed. We plot in Figure 2(c) the transmission spectrum at different asymmetrical parameter *θ* using measured refractive index values from Figure 2(b). It is clearly seen that with the decrease of *θ*, the resonance bandwidth becomes narrower and reaches a minimum value when *θ* decreases to 0, consistent with the behavior of Q factor described by the black solid points in Figure 2(a).

To observe the anti-crossing behavior in the strong coupling effect, the optical cavity resonance should be tuned over a broad band across the material transition. One typical approach to achieve the spectral tuning is to explore the spatial dispersion behavior of the QBIC resonance, i.e. the dependence of QBIC resonance on the incident angle [23]. Figure 2(d) presents the calculated resonance position (blue dots) and the corresponding Q factors (red squares) versus the transverse wave vector in the *x*-direction, respectively, which correspond to different incident angles in the *xz* plane. As one can see, with the increase of the transverse wave vector, the resonance experiences a significant red-shift with a slight increase of the Q factor. These results demonstrate that for a fixed geometry, changing the incident angle can indeed be used to tune the QBIC resonance. In our work, due to the limit of our measurement setup, we switch to a different approach of fixing the excitation at normal incidence while tuning the QBIC by controlling the PMMA thickness. Due to the refractive index of PMMA being larger than unity, the presence of a thin layer of PMMA on the structure causes a spectral shift of the QBIC resonance towards longer wavelengths. As the PMMA thickness gradually increases, the QBIC resonance will shift correspondingly and cross the vibrational resonance of PMMA. During this process, an anti-crossing phenomenon due to the strong coupling effect will be observed.

**Figure 3:**
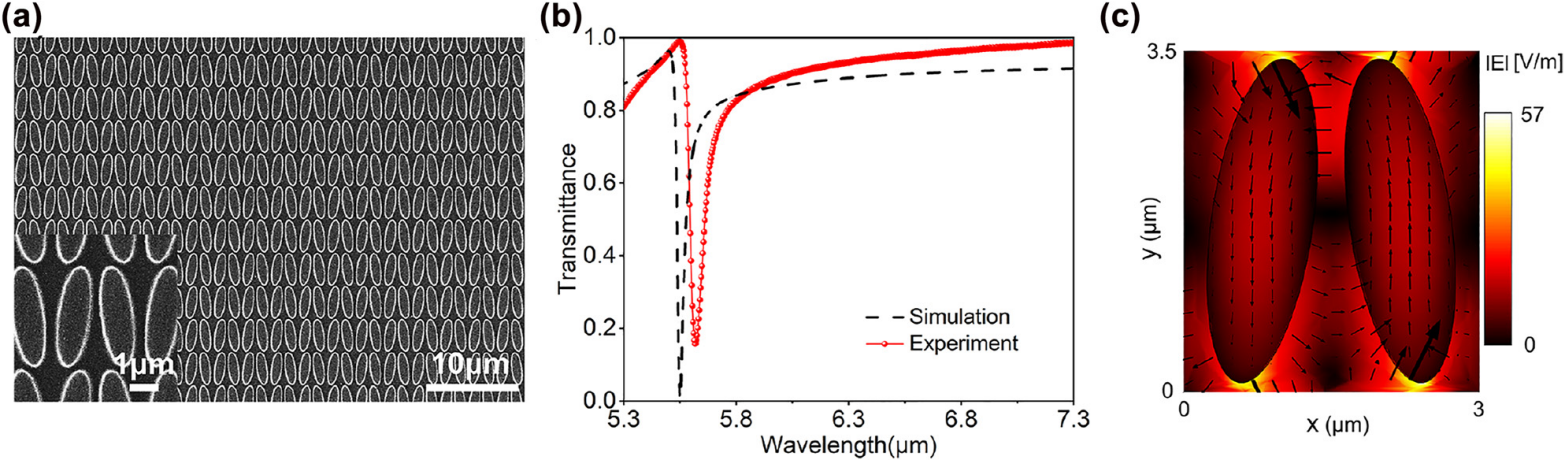
Characterization results of the metasurface. (a) An SEM image showing the top view of the fabricated elliptical disk zigzag metasurface array. (b) Transmission spectrum of the metasurface disk array with *θ* = 8°, where the black dashed line is extracted from numerical simulations while the red solid circles represent the experimentally measured results. (c) The amplitude as well as the vectorial distribution of the electric field across *xy* cross-sections of the elliptical disks.

Figure 3(a) shows a top-view scanning electron microscopy (SEM) image of the fabricated structure. The transmission spectrum of the sample was obtained using a Fourier transform infrared spectrometer (FTIR) under normal incidence at *x*-directional linear polarization. The results are shown by the red solid spheres in Figure 3(b), which agree reasonably with the numerical results in the black dashed line. Both results exhibit the same Fano-shaped profile and the same level of resonance bandwidth (0.039 and 0.056 μm for the numerical and experimental results, respectively, which correspond to the Q factors of 145 and 101). To keep the consistency of the unit, we use the wavelength measured in microns instead of following the convention of wavenumbers in the MIR. The relatively lower Q factor in the experimental results as well as the slight red-shift are attributed to the additional optical losses caused by the unsmooth sidewalls of the Ge disks and some other manufacturing defects. When simulating the near-field electric field distribution at the QBIC resonance, we find that a large field amplitude enhancement (the incident plane wave has an E field amplitude of 1 V/m) is generated near the apex of the long axis of the ellipse (Figure 3(c)). The highest local electric field enhancement occurs close to the outer boundary of the disks, facilitating a strong interaction between the QBIC resonance and external medium. From the vectorial distribution of the electric field in Figure 3(c), one can see that two electric dipoles with roughly opposite directions along the *y*-axis are excited, verifying the symmetry-protected characteristic of the QBIC resonance.

**Figure 4:**
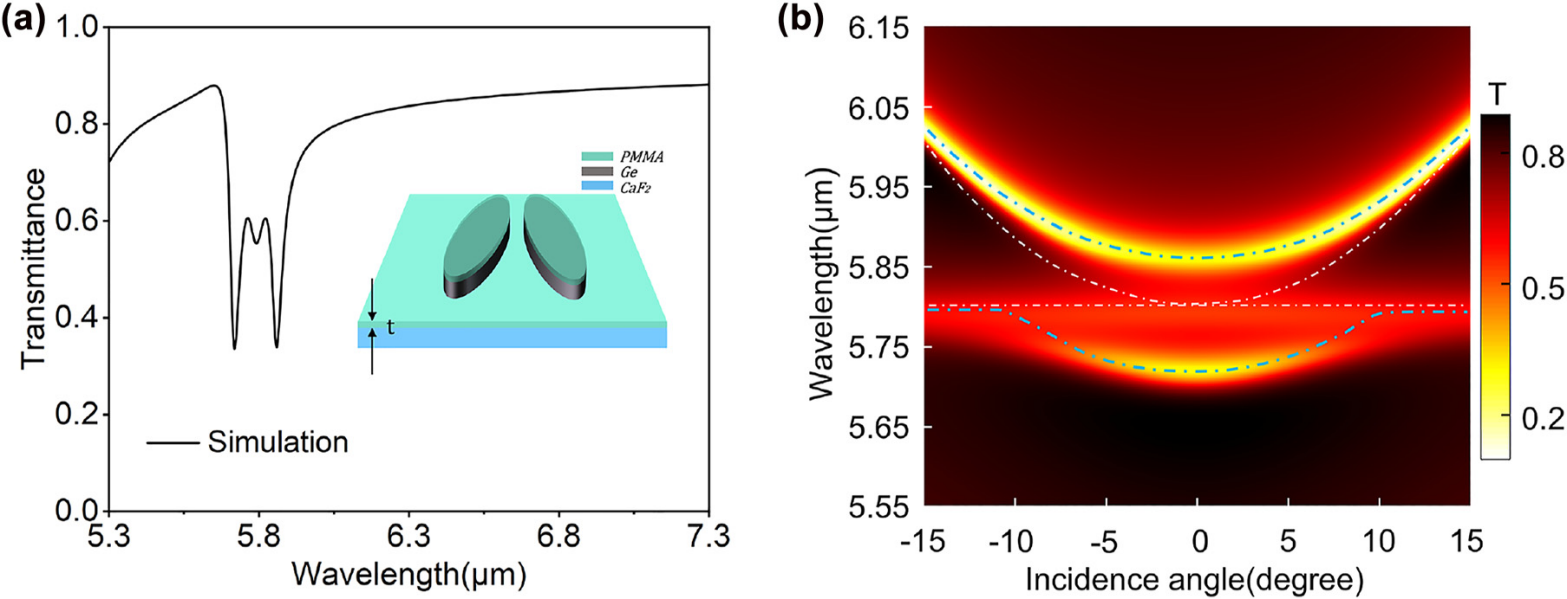
Generation of strong coupling and properties of the hybridization states. (a) Calculated transmission spectrum at normal incidence when a PMMA layer with the thickness of 78 nm is used. The strong coupling with resonance splitting phenomenon can be well observed. The inset illustrates a schematic diagram of the structure after spin-coating a thin layer of PMMA on top, where *t* represents the thickness of the PMMA layer. (b) The dispersion diagram calculated with the presence of the PMMA thin film (*t* = 78 nm).

In order to demonstrate the strong interaction between the QBIC resonance and the vibrational resonance of PMMA around 5.78 μm, we designed a Ge disk metasurface array with a slightly lower QBIC resonance wavelength, and then use PMMA of different thickness to tune the QBIC resonance to cross the PMMA vibration. We started from a structure with a calculated QBIC resonance of 5.55 μm, whose transmission spectrum is shown by the black dashed line in Figure 3(b). We first performed numerical investigations to shed light on the interactions between PMMA and QBIC resonances. In the simulations, since the number of molecules in the PMMA layer can be considered to be infinite, the coupling system behaves as a classical system. The PMMA molecular can then be described by a classical continuous medium with Lorentz permittivity [42–44]:

(2) $$\varepsilon_{\text{PMMA}}=\varepsilon_{\infty }-\frac{f_{0}\omega_{0}^{2}}{\omega^{2}-\omega_{0}^{2}-i\gamma \omega }$$

where the background relative permittivity of PMMA molecules is *ε*_*∞*_ = 2.2, the Lorentz resonance frequency is *ω*_*0*_ = 3.253 × 10^14^ rad/s, the strength coefficient *f*_*0*_ is 0.025 and the Lorentz damping rate *γ* is 3.0 × 10^12^ rad/s. All these parameters are obtained by fitting to previously reported experimental results [13,45]. As shown by a typical transmission spectrum in Figure 4(a), due to the strong interaction of the QBIC resonance with the vibrational resonance of PMMA, two hybrid photon-vibration modes emerge and manifest themselves as a peculiar double-resonance phenomenon in the transmission spectrum. When the PMMA thickness increases, the spectral position of the QBIC resonance shifts to achieve an exact matching with the PMMA vibrational resonance. From our calculations, we found that for our structure of bare Ge disk-pair array on CaF_2_, which exhibits the QBIC resonance around 5.55 μm, the optimal thickness of the PMMA layer is about 78 nm. With this optimal thickness, the QBIC resonance matches the PMMA vibration, leading to the largest splitting into the two hybridization resonances with the same strength, as shown by the calculated transmission spectrum in Figure 4(a). The small transmission dip between the two stronger resonances occurs at exactly the original vibrational resonance of PMMA, and is attributed to the absorption of PMMA directly on the CaF_2_ substrate.

In order to reveal the nature of the hybridization states, we plot in Figure 4(b) the transmittance through the Ge disk-pair metasurface array coated with the optimal PMMA thickness of 78 nm, calculated at different incident angles. A typical dispersive behavior of the coupled states in the strong coupling regime is shown. The blue dotted lines at the top and bottom represent the upper polariton (UP) and lower polariton (LP) modes from the strong coupling. The white curve and the horizontal dotted line represent the dispersion of QBIC and the vibrational resonance of PMMA molecules, respectively. The dispersion of QBIC is calculated by considering the background refractive index of PMMA molecules while eliminating its vibration. When the interaction between the QBIC resonance and the vibration of PMMA is considered, two hybrid states can be found at all the incident angles and the varying of the incident angle will lead to the transmission curves of both hybrid states repelling each other (corresponding to the two blue dashed lines in Figure 4(b)). At a normal incidence, where the QBIC matches exactly the original vibration resonance, the interaction between the two resonances reach the maximum and the spectral separation between the two branches is the minimum, where the value of Rabi splitting ℏ Ω_R_ can be determined.

**Figure 5:**
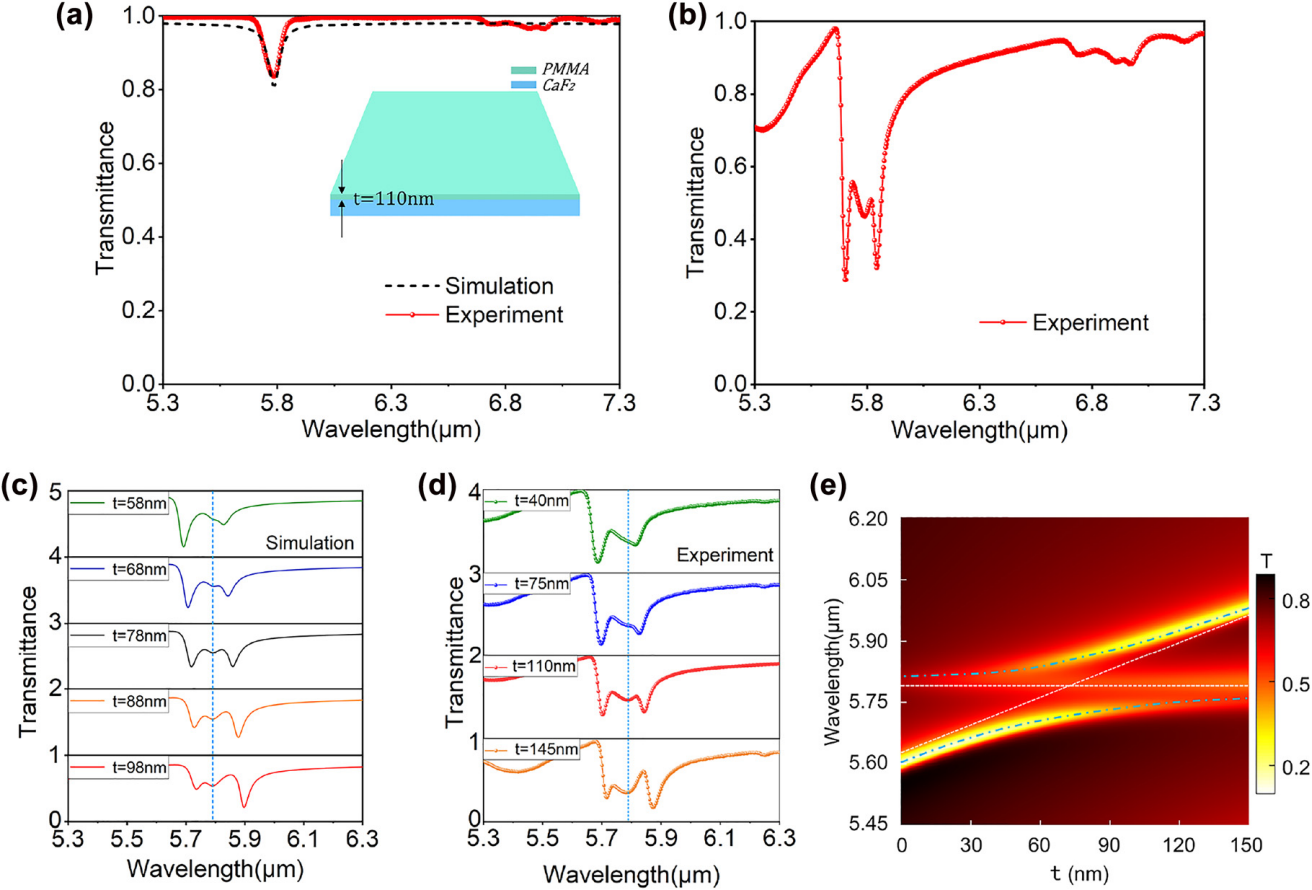
Theoretical simulation and experimental characterization of strong coupling under different PMMA film thicknesses. (a) Measured transmission spectrum of the PMMA layer with *t* = 110 nm on a bare CaF_2_ substrate, exhibiting a good agreement with the calculations based on the Lorentz model. (b) Transmission spectra measured when the sample was spin-coated with a 110 nm PMMA layer. (c) Calculated transmission spectra for different PMMA thicknesses (*t* = 58, 68, 78, 88, and 98 nm. (d) Experimentally measured transmission spectra for different PMMA thicknesses (*t* = 40, 75, 110, 145 nm). The blue vertical dashed lines in (c) and (d) represent the position of the vibrational resonance of PMMA molecules. (e) Transmission spectra of the hybrid structure with different PMMA thickness *t*.

Figure 5(a) presents the calculated transmission spectrum of a 110 nm-PMMA on a bare CaF_2_ substrate using the Lorentz model in Eq. (2), as well as the experimentally measured ones (red line) from FTIR. Both results exhibit the same level of absorption and bandwidth (0.07 and 0.078 μm for the numerical and experimental results, respectively). The good agreement between the experimental results from FTIR and the numerical calculations confirms the validity of the numerical model used in this work. The transmission spectrum of the disk array coated with 110 nm PMMA is measured and the results are shown in Figure 5(b). It can be clearly seen that the interaction between the QBIC resonance and vibrational resonance of PMMA molecules produces a double-resonance phenomenon, which are found at the wavelengths of 5.701 and 5.842 μm, respectively. The spectral separation between these two hybrid states, 0.141 μm, fully meets the strong coupling criteria [46] with the bandwidth of the QBIC resonance supported by the Ge disk array (0.056 μm from Figure 3(b)) and the absorption bandwidth of PMMA (0.078 μm from Figure 5(a)), confirming the strong-coupling characteristic of the composite structure. Subsequently, we numerically investigate the effect of the PMMA thickness on the strong coupling by simulating the transmission spectra of the Ge metasurface array with different PMMA thicknesses (58, 68, 78, 88, and 98 nm). The results are shown in Figure 5(c), where the blue dotted line indicates the vibrational resonance of PMMA. It can be seen that as the PMMA thickness increases, both hybrid states experience a redshift with an exchange of their relative strength. This phenomenon can be well verified in the experiment, as shown in Figure 5(d). Figure 5(e) presents a two-dimensional mapping of the transmittance through the coupled system at different PMMA thickness and excitation wavelength. The horizontal dotted line represents the vibrational resonance of PMMA, and the inclined white dotted line represents the QBIC resonance where only the real part of PMMA refractive index is considered. As can be seen from the upper and lower blue dotted lines which represent the UP and LP modes respectively, the anti-crossing behavior is quite significant. The results show that controlling the thickness of PMMA molecules is also an effective way to control the interaction between the two resonances.

Due to the presence of a dense and orderly distributed disk array on the CaF_2_ surface and a certain degree of roughness on the sidewall of the disks, the thickness of PMMA layer on the structure cannot be accurately controlled and have nonuniform distributions on the same sample (thicker within the gaps). As a result of those uncertainties, we observed in our experiments a strong coupling effect at *t* = 110 nm that matches the best with the simulated value at *t* = 78 nm (in Figure 4(a)). Furthermore, the extra loss caused by the roughness of the disk sidewalls leads to the reduction of Q-factor, which reduces the sensitivity of QBIC resonance on the thickness of PMMA layer. Nevertheless, the strong interaction between QBIC resonances and vibrations of PMMA can be easily observed, and the strong coupling between the two resonances is well demonstrated. As such, we do hope that our study will be considered as another important step towards developing applications based on light–matter interactions in the strong coupling regime.

## 3 Discussions and conclusion

In conclusion, we have demonstrated both numerically and experimentally the strong coupling phenomenon between the QBIC resonance of a metasurface consisting of all-dielectric nanostructures and the vibrational resonance of PMMA molecules in the MIR. Using PMMA layer of different thickness, the QBIC resonance was tuned across the vibration of PMMA and the typical features of strong coupling including mode splitting and anti-crossing are well observed. We note that in this work evaporated Ge with some dissipation loss is used as the dielectric material and an intermediate Q factor of 100 is experimentally achieved. Nevertheless, a room temperature strong coupling effect can still be easily achieved. If the amorphous Ge will be replaced by a crystalline structure or another dielectric, the overall properties of the QBIC resonance could be further improved and a more pronounced strong coupling can be expected. In general, our results demonstrate that the strong coupling between the QBIC and vibrations of organic materials is quite feasible in the infrared and may open up new prospects for novel applications based on the exotic properties of the hybrid photon-vibration states.

## Supplementary Material

Supplementary Material Details
