# Supplementary material for: Strong coupling between quasi-bound states in the continuum and molecular vibrations in the mid-infrared
Source: Nanophotonics. 2022 Aug 9;11(18):4221–9. doi: 10.1515/nanoph-2022-0311 (PMC11501134; doi:10.1515/nanoph-2022-0311)
Supplement: Supplementary file 1 — Supplementary Material Details [file j_nanoph-2022-0311_suppl_001.docx]

Supporting Information

Strong coupling between quasi bound states in the continuum and molecular vibrations in the mid-infrared

Kaili Sun^1^, Min Sun^1^, Yangjian Cai^1^, Uriel Levy^2^ and Zhanghua Han^1, *^

^1^Shandong Provincial Key Laboratory of Optics and Photonic Devices, Center of Light Manipulation and Applications, School of Physics and Electronics, Shandong Normal University, Jinan 250358, China

^2^ Department of Applied Physics, and the center for Nanoscience and Nanotechnology, The Hebrew University of Jerusalem, Jerusalem, Israel

*Email: zhan@sdnu.edu.cn

**Numerical simulations.**

The properties of the QBIC modes supported by the metasurface structure, including both the resonance frequencies and the corresponding quality factors, are evaluated using the eigen-frequency analysis (implemented in a commercial software of Comsol Multiphysics), combining the Floquet period boundary conditions in the lateral directions with perfectly matched layers (PML) in the *z*-direction. The transmission spectra are calculated by using S parameters with the port boundary conditions.

In all the simulations, the dielectric constant of the CaF_2_ substrate is obtained by fitting the tabulated experimental data [1] into polynomials described by the formula:

$\varepsilon_{{CaF}_{2}}=1.33973+\frac{0.69913\lambda^{2}}{\lambda^{2}-{0.09374}^{2}}+\frac{0.11994\lambda^{2}}{\lambda^{2}-{21.18}^{2}}+\frac{4.35181\lambda^{2}}{\lambda^{2}-{38.46}^{2}}$ (1)

where λ is the wavelength. In contrast, the permittivity of PMMA is modeled using a Lorentz oscillating model shown as follows [2]:

$\varepsilon_{PMMA}=\varepsilon_{\infty}-\frac{f_{0}\omega_{0}^{2}}{\omega^{2}-\omega_{0}^{2}-i\gamma\omega}$ (2)

where the background relative permittivity of PMMA molecules is *ε_∞_* = 2.2, the Lorentz resonance frequency is *ω_0_* = 3.253×10^14^ rad/s, the strength coefficient *f_0_* is 0.025 and the Lorentz damping rate *γ* is 3.0×10^12^ rad/s. All these parameters are obtained by fitting to previously reported experimental results  [3] [4].

The geometrical parameters of the zigzag array of Ge elliptical disks are as follows: h=300nm is the height of the Ge disks, *P_x_*=3μm and *P_y_*=3.5μm are the array period, and *a*=3.35μm and *b*=1.03μm represent the long and short axis of the elliptical disks, respectively. The two Ge disks within one unit cell has a center-to-center distance of 1.4μm.

**Sample fabrication.**

We prepared the sample on a double-side polished CaF_2_ substrate. First, a 300nm-thick Ge film was deposited onto the CaF_2_ substrate using electron beam evaporation (DETECH, DE500C) at a deposition rate of 1Å/s and the vacuum chamber pressure of 9×10^-7^Torr. After that, the PMMA resist (950 A4 from KayakuAM) was spin-coated onto the sample at 3500 *rpm* for 60s, followed by a baking at 180℃ for 90s to obtain a 200 nm thick PMMA layer. For the next step, the designed elliptical disk-pair patterns were exposed onto the PMMA layer by a 50KV electron beam lithography (EBL) system (Raith Voyager). After developing (1:3 MIBK-to-IPA for 60s followed by a 30s rinse in IPA), 30nm Al was evaporated onto the sample and the lift-off process was used to transfer the pattern from PMMA to Al layer. The Al pattern worked as a mask to obtain the Ge disk array in a subsequent inductive coupled plasma enhanced reaction ion etching (ICP-RIE) process (Plasma Pro 80 Cobra), and finally the residual Al was completely removed by soaking in NaOH solution for 30s and cleaned with deionized water. PMMA layers of different thicknesses was obtained by first diluting the EBL resist (950 A2 from KayakuAM) into anisole and then spin coating the mixture onto the metasurface at different rotational speeds, followed by a standard baking at 180℃ for 90s.

To prepare thin PMMA layers on top of the disk array, PMMA layers of different thicknesses was obtained by first diluting the EBL resist (950 A2 from KayakuAM) into anisole and then spin coating the mixture onto the metasurface at different rotational speeds. The thickness of the PMMA layer is controlled by both the dilution ratio and by the rotation speed of the spin-coating process. After the spin-coating, all the samples are baked at 180℃ for 90s to remove the remaining anisole. Using an optical ellipsometer and the reference sample of PMMA coated on a bare CaF_2_ substrate at the same rotation speed, we can determine the PMMA thickness.

**Optical measurements.**

The morphology of the top surface of the sample was characterized by the scanning electron microscope (Sigma 500, Zeiss), and the images show that the structure matches well with the design, thus verifying the fabrication accuracy. All the transmission spectra for all the samples including the PMMA layer on CaF_2_ substrate, the metasurface structure with and without PMMA layers, were measured using a Fourier transform infrared spectrometer (FTIR) (Bruker Vertex 80-V) equipped with a home-made linear polarizer working in the MIR composed of subwavelength gold nanowire gratings on a CaF_2_ substrate. The data on the MIR refractive index of the Ge and its thickness are derived from measurements performed by an Ellipsometer working in the MIR (IR-VASE Mark II from J. A. Woollam).

References:

[1]. H. H. Moore, *Refractive Index of Alkaline Earth Halides and Its Wavelength and Temperature Derivatives*, J. Phys. Chem. Ref. Data **9**, 161 (1980).

[2]. B. Lahiri, S. G. McMeekin, R. M. De La Rue, and N. P. Johnson, *Enhanced Fano Resonance of Organic Material Films Deposited on Arrays of Asymmetric Split-Ring Resonators (A-SRRs)*, Opt. Express **21**, 9343 (2013)

[3]. W. Wan, X. Yang, and J. Gao, *Strong Coupling between Mid-Infrared Localized Plasmons and Phonons*, Opt. Express **24**, 12367 (2016).

[4]. F. Cheng, X. Yang, and J. Gao, *Ultrasensitive Detection and Characterization of Molecules with Infrared Plasmonic Metamaterials*, Sci. Rep. **5**, 14327 (2015).
